# First insights into the genotype–phenotype map of phenotypic stability in rye

**DOI:** 10.1093/jxb/erv145

**Published:** 2015-04-06

**Authors:** Yu Wang, Michael Florian Mette, Thomas Miedaner, Peer Wilde, Jochen C. Reif, Yusheng Zhao

**Affiliations:** ^1^Department of Breeding Research, Leibniz Institute of Plant Genetics and Crop Plant Research (IPK), D-06466 Gatersleben, Germany; ^2^State Plant Breeding Institute, University of Hohenheim, 70593 Stuttgart, Germany; ^3^KWS LOCHOW GMBH, 29296 Bergen, Germany

**Keywords:** Genetic architecture, genomic selection, marker-assisted selection, phenotypic stability, rye, yield stability.

## Abstract

Improving phenotypic stability of crops is pivotal for coping with the detrimental impacts of climate change. The goal of this study was to gain first insights into the genetic architecture of phenotypic stability in cereals. To this end, we determined grain yield, thousand kernel weight, test weight, falling number, and both protein and soluble pentosan content for two large bi-parental rye populations connected through one common parent and grown in multi-environmental field trials involving more than 15 000 yield plots. Based on these extensive phenotypic data, we calculated parameters for static and dynamic phenotypic stability of the different traits and applied linkage mapping using whole-genome molecular marker profiles. While we observed an absence of large-effect quantitative trait loci (QTLs) underlying yield stability, large and stable QTLs were found for phenotypic stability of test weight, soluble pentosan content, and falling number. Applying genome-wide selection, which in contrast to marker-assisted selection also takes into account loci with small-effect sizes, considerably increased the accuracy of prediction of phenotypic stability for all traits by exploiting both genetic relatedness and linkage between single-nucleotide polymorphisms and QTLs. We conclude that breeding for crop phenotypic stability can be improved in related populations using genomic selection approaches established upon extensive phenotypic data.

## Highlight

Yield stability in rye is controlled by many QTLs with small effects. This is in contrast to phenotypic stability of several quality traits, which are influenced by some large-effect QTLs.

## Introduction

The primary goal in plant breeding is to identify high-yielding genotypes combining excellent quality with pronounced resistance to abiotic and biotic stresses. With stress levels increasing due to climate change (Schmidhuber and Tubiello, 2007), enhanced phenotypic stability is becoming more important. Stability can be defined either as static or dynamic phenotypic stability (Becker and Léon, 1988). Static phenotypic stability refers to the ability of a genotype to realize a constant performance independent of the variation of environmental conditions. Static phenotypic stability, however, for traits such as grain yield, is often associated with relatively low performance (Lin *et al.*, 1986; Becker and Léon, 1988). In this case, dynamic phenotypic stability concepts describing the ability of a genotype to respond to improved agronomic conditions of an environment with increased performance are considered to be more relevant. Dynamic stability can be estimated by Shukla’s (1972) stability variance, or by Wricke’s (1962) ecovalence, which provide a measure of genotype stability based on estimates of genotype and environment interaction variance corresponding to an individual genotype.

Large-scale phenotyping is required to precisely determine yield stability, with estimates for the number of required environments ranging from 10 (Becker, 1987) to 200 (Piepho, 1998). As phenotyping is highly demanding on resources, genomics-assisted breeding is a promising tool for the improvement of phenotypic stability. Substantial progress was made to elucidate the genetic architecture of agronomic and quality traits by marker-assisted selection focusing on major quantitative trait loci (QTLs; Lande and Thompson, 1990; Dekkers and Hospital, 2002; Collard and Mackill, 2008). In addition, genomic selection approaches have been implemented to improve breeding for complex agronomic traits by also exploiting minor-effect QTLs as well as genetic relatedness (Zhong *et al.*, 2009; Albrecht *et al.*, 2011; Heffner *et al.*, 2011; Rutkoski *et al.*, 2012; Windhausen *et al.*, 2012; Zhao *et al.*, 2012). Knowledge of the genetic architecture underlying phenotypic stability, however, is very limited, and so far there has been only one association-mapping study performed in barley (Kraakman *et al.*, 2004). Moreover, the potential of whole-genome prediction approaches for phenotypic stability have not been investigated.

In Central Europe, rye is commonly improved by hybrid breeding based on a cytoplasmic male sterility system (Fischer *et al.*, 2010) and is used for bread making, feed, and as a valuable resource for renewable energy (Geiger and Miedaner, 2009). Important quality traits are falling number as well as pentosan and protein content, which determine the baking quality and feed value (Geiger and Miedaner, 1999). In addition, test weight is often considered an indirect criterion for starch content, which also influences milling and baking quality and ethanol yield. Rye possesses extraordinary resistance against frost stress (Li *et al.*, 2011) and exhibits high performance even in marginal environments (Geiger and Miedaner, 2009). Therefore, it is considered a model species for elucidating abiotic stress resistance (Martis *et al.*, 2013), and in line with this, rye can be considered a model species suitable for studying the genetic architecture of phenotypic stability.

For our study, we evaluated two segregating F_3:4_ rye test-cross populations connected through one common parent, each comprising 220 lines, for grain yield, thousand kernel weight, test weight, falling number, and both protein and pentosan content in up to 16 environments, including drought-stress environments (Hübner *et al.* 2013). Interestingly, we observed that static phenotypic stability was only marginally associated with low performance. Thus, static phenotypic stability is an interesting trait for breeding in rye. The extensive phenotypic information was combined in a linkage-mapping study with genome-wide molecular marker data with the aim of searching for large-effect QTLs underlying phenotypic stability. We observed an absence of major-effect QTLs for yield stability and its component thousand kernel weight stability. Nevertheless, performance of static yield stability could be predicted with moderate to high accuracies by applying genome-wide selection approaches, pointing to a rather complex genetic architecture. By contrast, large and stable QTLs were found for static phenotypic stability of the quality traits test weight, soluble pentosan content, and falling number. In contrast to marker-assisted selection, applying genome-wide prediction approaches again considerably increased accuracies of prediction of phenotypic stability for all quality traits.

## Materials and methods

### Plant material and field experiments

The plant material and field experiments were described in detail by Miedaner *et al.* (2012), Hübner *et al.* (2013), and Wang *et al.* (2014). Briefly, we used three elite winter rye (*Secale cereale* L.) inbred lines (Lo90-N, Lo115-N, and Lo117-N) as parents for this study. Two segregating F_3:4_ populations, POP-A (Lo90-N × Lo115-N) and POP-B (Lo115-N × Lo117-N), were generated. From each population, we randomly selected 220 F_3:4_ progenies, which were used to pollinate a common cytoplasmic male sterile (CMS) single-cross tester.

The test-cross families were evaluated in two years (2010 and 2011) at five locations [Wohlde (WOH), Germany, N52.8°, E10.0°, 80 m above sea level, loamy sand soil texture; Beckedorf (BEK), Germany, N52.5°, E10.3°, 80 m above sea level, loamy sand soil texture; Petkus (PET), Germany, N51.6°, E13.2°, 130 m above sea level, sandy soil texture; Hohenheim (HOH), N48.4°, E9.1°, 400 m above sea level, loamy soil texture; Walewice (WAL), Poland, N52.6°, E19.4°, 184 m above sea level, heavy loam soil texture] with different water regimes (WOH-2010-i, WOH-2010-n, WOH-2011-m, BEK-2010-m, BEK-2011-i, BEK-2011-n, PET-2010-i, PET-2010-n, PET-2011-i, PET-2011-n, HOH-2010-m, HOH-2011-m, WAL-2010-i, WAL-2010-n, WAL-2011-i, and WAL-2011-n, where ‘i’ refers to irrigation in drought stress environments, ‘n’ denotes non-irrigation in drought stress environments, and ‘m’ refers to no drought stress environments). The respective location × year × irrigation level combinations were denoted as environments throughout the study. The two test-cross populations were evaluated in each environment using an incomplete 24×10 alpha design with two replications. Data for grain yield (dt ha^–1^), thousand kernel weight (g), test weight (g), falling number (s), protein content (%), and soluble pentosan content (%) were obtained as outlined in detail by Miedaner *et al.* (2012) and Wang *et al.* (2014). Briefly, protein content and soluble pentosan content were determined by near-infrared reflectance spectroscopy (NIRS) recorded with a Bruker MPA FT-NIRS instrument (Bruker Optics, Ettlingen, Germany) in reflectance mode over a range from 850 to 2500nm. The samples were scanned twice in duplicate repacking using two different Petri dishes of 8.7cm diameter and 1cm height as sampling cups on a rotating device with on average 32 scans in 10 and 2 spectra per sample. The NIRS calibrations were developed based on up to 330 samples of the two populations using reference values for protein content and soluble pentosan content determined as outlined in detail elsewhere (Jürgens *et al.*, 2012). Prediction models were established with OPUS Software version 6.5 (Bruker Optic GmbH, Ettlingen, Germany) using a modified partial least-squares procedure with a validation and scatter correction (SNV) of the spectra. Spectra were tested as original and first derivatives. The robustness of the calibrations has been tested in an independent validation set of around 100 samples. The final calibration models were applied on near-infrared reflectance spectra collected from the field trials to predict phenotypic values per field plot.Test weight (g) and falling number (s) were phenotyped according to internationally standardized (AACC 55-10 and AACC 56-81B) methods, respectively.

### Phenotypic data analysis

Best linear unbiased estimates (BLUEs) for test-cross progenies across environments were determined by the restricted maximum likelihood method using ASReml version 3.0 (Gilmour *et al.*, 2009) based on a two-step approach. In step one, the following model was applied:

$$y_{Env}=\;l_{n}µ+G\alpha_{\text{G}}+R\alpha_{\text{R}}+B\alpha_{Β}+e$$ (1)

where *y*_Env_ represents BLUEs of single environments, 1_n_ denotes a vector with length n (n is the number of genotypes multiplied by the number of replications), *µ* represents the overall mean, *G* refers to a design matrix of genotypes, *α*_G_ is an N-vector of the genotype effects with length N equal to the number of genotypes, *R* corresponds to a design matrix for replications, *α*_R_ makes reference to a vector of the replication effects, *B* stands for a design matrix of the blocks, *α*_B_ refers to a vector of the block effects, and *e* is a residual term. Genotype effects were treated as fixed while the other factors included in model (1) were treated as random. BLUEs of test-cross progenies within each environment were estimated in step one. BLUEs of test-cross progenies across different environments were estimated in step two as follows:

$$y_{Envs}=\;l_{k}µ+G\alpha_{\text{G}}+E\alpha_{Env}+F\alpha_{\text{F}}+e$$ (2)

where *y*_Envs_ refers to the BLUEs across all the environments, 1_k_ is a vector with the length k equal to the number of genotypes multiplied by the number of environments, *E* denotes a design matrix of environments, *α*_Env_ represents a vector of environment effects, *F* is a design matrix of genotype × environment interactions, *α*_F_ represents a vector of interaction effects, and *e* a residual term. We kept genotype effects fixed, and treated both environment and interaction effects as random. Cluster analysis of environments was performed based on 1 minus the correlation coefficient of BLUEs of genotypes among all pairs of environments. Heritability was estimated on an entry-mean basis (*h* ^2^) as outlined in detail elsewhere (Wang *et al.*, 2014).

### Estimation of phenotypic stability parameters

According to Roemer (1917), environmental variance (Var) of an individual genotype *i* is estimated as:

$${\text{Var}}_{\text{i}}=\;\frac{\sum_{j}{\left(y_{ij}-{\bar{y}}_{i.}\right)}^{2}}{N_{E}}$$ (3)

where *y*_*ij*_ refers to the BLUEs of genotype *i* in environment *j* of a set of N_E_ environments and ${\bar{y}}_{i.}$ denotes the marginal means of BLUEs of genotype *i* in the N_E_ environments. A smaller environmental variance indicates a higher phenotypic stability according to the static phenotypic stability concept (Becker and Léon, 1988). According to the Eberhart-Russell model (Eberhart and Russell, 1966), the regression coefficient (β), measuring the sensitivity of an individual genotype to the varying environments, was estimated as:

$$\beta_{i}\;=\frac{\sum_{j}\;y_{ij}({\bar{y}}_{.j}-\;{\bar{y}}_{..})}{{\left({\bar{y}}_{.j}-\;{\bar{y}}_{..}\right)}^{2}}$$ (4)

with ${\bar{y}}_{.j}$ referring to the marginal mean of BLUEs of all genotypes in the *j-th* environment and ${\bar{y}}_{..}$. denoting the overall mean. Smaller regression coefficients indicate higher phenotypic stability according to the static phenotypic stability concept (Eberhart and Russell, 1966). Additionally, deviation variance (Dev) was estimated as a measure of dynamic stability as:

$$Dev_{\text{i}}=\frac{1}{N_{E}-2}\left[\underset{j}{\sum }{\left(y_{ij}-\;{\bar{y}}_{i.}\right)}^{2}-\beta_{i}{}^{2}\underset{j}{\sum }{\left({\bar{y}}_{.j}-\;{\bar{y}}_{..}\right)}^{2}\right]$$ (5)

A smaller deviation variance indicates a higher stability according to the dynamic phenotypic stability concept (Eberhart and Russell, 1966). Spearman’s rank correlation coefficient was calculated between six traits of interest and dynamic and static phenotypic stability parameters for each population. We used the approach based on the Spearman’s rank correlation, because it is less sensitive to outliers as compared to the method based on Pearson’s moment correlations. Moreover, we applied the resampling strategy proposed by Mühleisen *et al.* (2014) and estimated the heritability of phenotypic stability parameters by additionally projecting the expected heritability for a situation of phenotypic evaluation in up to 30 environments.

### Marker-assisted selection for phenotypic stability

In total 440 F_3:4_ lines from Pop-A and Pop-B were genotyped with up to 81 simple sequence repeats (SSRs), 732 single-nucleotide polymorphisms (SNPs), and 900 diversity array technology (DArT) markers. Details of the marker data used in our study have been outlined elsewhere (Miedaner *et al.*, 2012; Wang *et al.*, 2014) (Supplementary Table S1) and were used in combination with the environmental variance (Var), regression coefficient (β), and logarithmic transformations (base-*e* logs) of deviation variance (Dev_ln_) in classical bi-parental linkage mapping. The conditional probabilities of QTL genotypes have been estimated with the software package PlabQTL (Utz and Melchinger, 1996). Marker-assisted selection was carried out using PLABQTL (Utz and Melchinger, 1996) adopting composite interval mapping (CIM) based on the regression approach (Haley and Knott, 1992) in combination with the use of cofactors (Jansen and Stam, 1994; Zeng, 1994). Cross-validation was performed using PLABQTL (Utz and Melchinger, 1996) and accuracies of prediction were calculated as:

$$r_{g}=\sqrt{\frac{R_{CV}^{2}}{h^{2}}}$$ (6)

where *R* ^2^_CV_ is the percentage of phenotypic variance of the validation set explained by the identified QTL and *h* ^2^ denotes the heritability of each phenotypic stability parameter. Moreover, we recorded the percentage of cross-validation runs in which QTLs were detected.

### Genomic selection

We used ridge regression best linear unbiased prediction (RR-BLUP) (Whittaker *et al.*, 2000) implemented as outlined in detail in our companion study (Wang *et al.*, 2014) to perform genomic selection. The RR-BLUP model has the form:

$$\text{y}=1_{n}\mu +\text{Xg}+\text{e}$$ (7)

where y is the vector of the estimated phenotypic stability parameters, 1_n_ denotes the vector of 1s, μ refers to the overall mean, g denotes the vector of marker effects, X stands for the corresponding design matrix (Supplementary Tables S2 and S3) and e corresponds to the residual term of the model. The relevant parameters for RR-BLUP were estimated based on the mixed model equations outlined by Wang *et al.* (2014).

### Cross-validation

We applied 5-fold cross-validation, where data sets were split into an estimation set for estimating marker effects, and a test set, where the Pearson’s correlation coefficients (*r*_MP_) between an observed phenotypic stability parameter and its predicted values based on the determined marker effects were calculated. Accuracy of prediction was estimated by standardizing with the square root of the heritability, *r*_g_ = *r*_MP_ / *h* (Lande and Thompson, 1990; Dekkers, 2007; Albrecht *et al.*, 2011). Sampling of estimation and test sets was repeated 5000 times in each cross-validation scheme.

## Results

### Heritabilities and phenotypic stability parameters vary for six agronomic traits

The quality traits protein content and soluble pentosan content were determined using NIRS. For both traits, stable and accurate prediction models have been developed, with coefficients of determination in the validation set of 0.98 for protein content and 0.74 for soluble pentosan content (Table 1). Consequently, indirect measurement of the traits should not hamper further QTL and genomic selection analyses.

**Table 1.** Accuracy of prediction for developed NIRS calibrations of protein content and soluble pentosan content

Model	Calibration				Validation					
	*N*_*C*_	*R*_*C*_	$R_{C}^{2}$	SE	*N*_*V*_	Bias	*R*_*V*_	$R_{V}^{2}$	SE	SD
PC (%)	330	0.99	0.98	0.23	108	0.01	0.99	0.98	0.31	2.07
SPC (%)	321	0.91	0.82	0.18	107	–0.02	0.86	0.74	0.22	0.43

*N*_*C*_ and *N*_*V*_ denote sample sizes for calibration and validation, respectively; *R*_*C*_ and *R*_*V*_ refer to the correlation coefficients of calibration and validation, respectively; $R_{C}^{2}$ and $R_{V}^{2}$. represent coefficient of determination of calibration and validation, respectively; SE is standard error of calibration and validation, respectively; SD denotes the standard deviation within the validation set; PC, protein content; SPC, soluble pentosan content.

**Fig. 1.**
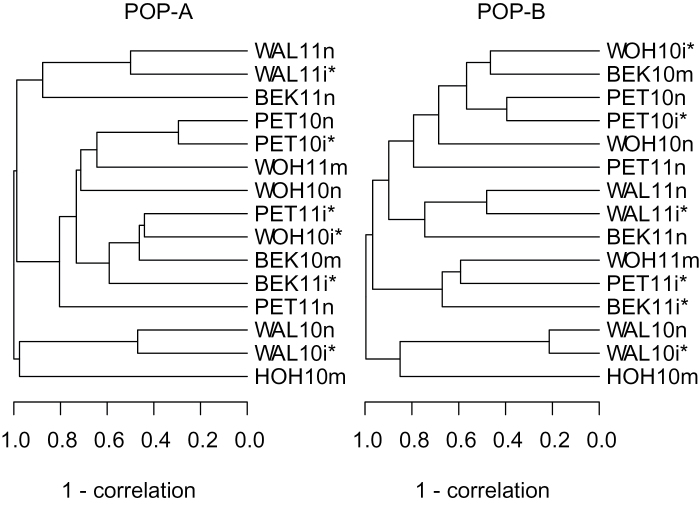
Dendrogram of cluster analysis including 15 environments (location × year × water availability) evaluated for grain yield for POP-A and POP-B. The cluster analysis is based on one minus the correlation coefficients among best linear unbiased estimates of single environments (for nomenclature of the environments see Material and methods). The asterisk denotes environments with severe drought stress leading to a reduction in average grain yield of more than 15%.

We evaluated 440 F_3:4_ test-cross progenies of two segregating rye populations in up to 16 diverse environments in Central Europe. The diversity of growing conditions is reflected in values of the variance component of environments *σ*_*E*_^2^ that are consistently significantly (*P* < 0.01) larger than zero (Table 2). The diversity of testing conditions is also portrayed in the complex grouping of testing environments based on the best linear unbiased estimates of the progenies for grain yield (Fig. 1) as well as for the examined quality traits (Supplementary Figure S1). The clustering of locations was not stable across years and also differed between both populations, clearly indicating that subgrouping of locations in homogeneous sets is impossible. Consequently, the large-scale phenotyping in diverse environments conducted in our study provides a unique data base in order to study the genetics of phenotypic stability across location, year, and water availability.

**Table 2.** Estimates of variance components and heritability on an entry-mean basis (*h* ^2^) for grain yield, thousand kernel weight, test weight, falling number, protein content, and soluble pentosan content of POP-A and POP-B

Trait	Mean	Range	$\sigma_{E}^{2}$	$\sigma_{G}^{2}$	$\sigma_{G}^{2}{}_{\times E}$	$\sigma_{Eff.Error}^{2}$	*h* ^2^
POP-A							
GY	72.8	54.5–81.3	278.72**	3.34**	8.91**	5.07	0.81
TKW	33.7	30.4–36.3	38.26**	1.11**	1.02**	0.33	0.93
TW	69.4	67.1–71.5	8.69**	0.52**	0.37**	0.86	0.95
FN	169.1	146.3–190.5	5948.46**	51.8**	172.04**	193.85	0.74
PC	9.9	9.2–10.6	0.68**	0.04**	0.14**	0.07	0.68
SPC	2.3	2.2–2.6	0.12**	0.002**	0.01**	0.01	0.65
POP-B							
GY	68.5	53.8–88.1	263.37**	4.13**	9.10**	4.81	0.84
TKW	32.6	29.4–36.5	37.72**	1.00**	0.79**	0.23	0.94
TW	70.3	66.6–72.6	11.05**	0.80**	0.49**	1.19	0.96
FN	179.8	155.7–202.8	4097.86**	48.41**	82.11**	192.45	0.83
PC	10.2	9.4–11.1	0.79**	0.07**	0.13**	0.05	0.83
SPC	2.3	2.1–2.5	0.14**	0.004**	0.01**	0.01	0.76

GY, grain yield (dt ha^–1^); TKW, thousand kernel weight (g); TW, test weight (g); FN, falling number (s); PC, protein content (%); SPC, soluble pentosan content (%); *σ*_*G*_^2^ refers to the genotypic variance; *σ*_*G×E*_^2^ represents the interaction variance between genotype and environment; σ_Eff_^2^, error denotes the variance of effective error; **, significantly different from zero with *P* < 0.01.

For the six traits and both populations, we also consistently observed genotypic variance components *σ*_*G*_^2^ significantly (*P* < 0.01) larger than zero (Table 2). The variance components of genotype × environment interactions *σ*_*G×E*_^2^ for POP-A and POP-B were also significantly (*P* < 0.01) larger than zero for all traits and exceeded twice the genotypic variances in the case of grain yield. The broad variation observed for best linear unbiased estimates of the 440 F_3:4_ test-cross progenies (Supplementary Table S4) resulted in high heritabilities on an entry-mean basis (*h* ^*2*^) for the six traits under consideration.

**Fig. 2.**
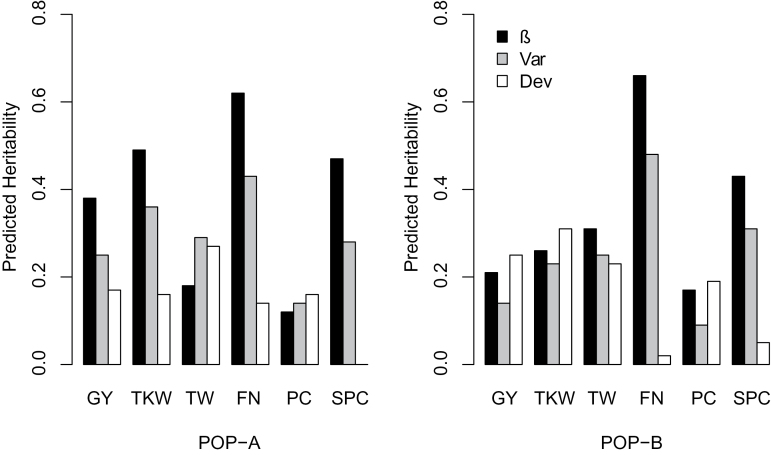
Estimates of predicted heritability of the stability parameters β, Var, and Dev for grain yield (GY, dt ha^–1^), thousand kernel weight (TKW, g), test weight (TW, g), falling number (FN, s), protein content (PC, %), and soluble pentosan content (SPC, %) for POP-A and POP-B.

In contrast, heritabilities estimated for the three phenotypic stability parameters, Var and β for static and Dev for dynamic stability, were substantially smaller (Fig. 2). Moreover, we observed a clear tendency for heritabilities estimated for the two static phenotypic stability parameters, Var and β, to be larger than heritabilities found for the dynamic phenotypic stability parameter Dev. The observed discrepancy between dynamic and static phenotypic stability measures suggests that the latter class of parameters is particularly interesting for studies elucidating their underlying genetic architecture in detail.

### Increased phenotypic stability is correlated with impaired grain yield and quality only in a minority of cases

A rye ideotype would combine excellent trait performance with high phenotypic stability. As these two features have previously been discussed as mutually exclusive, we inspected the associations between trait performance and the three phenotypic stability parameters (Table 3). For 25% of the associations between trait performance and phenotypic stability, we detected significant (*P* < 0.05) but low correlation coefficients in the undesired direction. In contrast, for 19% of the observations we found significant (*P* < 0.05) correlations in the desired direction, while for 56% of the cases correlation coefficients were not significantly (*P* > 0.05) different from zero. Consequently, breeding rye hybrids combining excellent trait performance with high phenotypic stability is impaired by contrasting pleiotropic effects or linkage among favourable alleles in repulsion phase only in the minority of cases.

**Table 3.** Estimates of correlation coefficients between different phenotypic stability parameters and best linear unbiased estimates for grain yield, thousand kernel weight, test weight, falling number, protein content, and soluble pentosan content POP-A and POP-B

Stability parameter	POP-A						POP-B					
	Number of environments						Number of environments					
	15		16	11	10		15		16	11	10	
	GY	TKW	TW	FN	PC	SPC	GY	TKW	TW	FN	PC	SPC
β	0.00	0.25**	–0.19*	0.08	0.26**	0.01	–0.05	0.13*	–0.14*	0.35**	0.02	0.25**
Var	0.02	0.25**	–0.24**	0.12	0.18**	0.03	–0.08	0.14*	–0.15*	0.35**	0.02	0.26**
Dev	0.11	0.00	–0.08	0.12	–0.09	0.06	–0.13*	0.17**	–0.02	–0.02	–0.02	0.11

GY, grain yield (dt ha^–1^); TKW, thousand kernel weight (g); TW, test weight (g); FN, falling number (s); PC, protein content (%); SPC, soluble pentosan content (%); * and **, significant at the 0.05 and 0.01 probability levels, respectively.

### Linkage mapping revealed a low number of stable QTLs for phenotypic stability

We performed linkage mapping in combination with 5-fold cross validations and observed very low accuracies of prediction of dynamic phenotypic stability Dev with a maximum value amounting to 0.13 for thousand kernel weight (Table 4). In contrast, accuracies of prediction for static phenotypic stability parameters Var and β with marker-assisted selection were for most traits higher compared to accuracies of prediction for Dev. A closer look at the cross-validated results revealed stable marker–trait associations, defined as QTLs detected in >50% of the reference populations for the quality traits test weight, falling number, and soluble pentosan content alone. Interestingly, the QTL identified for static phenotypic stability for falling number on chromosome 6 was detected in both populations. The other stable QTLs were identified exclusively in POP-B even under a reduced significant threshold of a LOD value of 2.5, except for the soluble pentosan content QTL on chromosome 7 for the parameter β, which was also detected under a reduced LOD threshold in POP-A.

**Table 4.** Cross-validated standardized accuracy of prediction for marker-assisted selection of three phenotypic stability parameters of six traits

Traits	*r*_g_β	QTL_β_	Chr. / Pos. (*R* ^2^)	*r*_g_Var	QTL_Var_	Chr. / cM (*R* ^2^)	*r*_g_Dev_ln_	QTL_ln_Dev_	Chr. / cM (R^2^)
POP-A									
GY	0.19	0	–	0.25	0	–	0.03	0	–
TKW	0.22	0	–	0.19	0	–	0.00	0	–
TW	0.13	0	–	0.26	0	–	0.00	0	–
FN	0.51	1	Chr. 6 / 24 cM (0.21)	0.58	1	Chr. 6 / 28 cM (0.16)	0.00	0	–
PC	0.05	0	–	0.04	0	–	0.00	0	–
SPC	0.03	0	–	0.00	0	–	0.00	0	–
POP-B									
GY	0.37	0	–	0.59	0	–	0.00	0	–
TKW	0.49	0	–	0.48	0	–	0.13	0	–
TW	0.57	1	Chr. 1 / 34 cM (0.12)	0.08	0	–	0.07	0	–
FN	0.41	1	Chr. 6 / 40 cM (0.16)	0.50	1	Chr. 6 / 40 cM (0.17)	0.00	0	–
PC	0.02	0	–	0.00	0	–	0.04	0	–
SPC	0.26	1	Chr. 7 / 90 cM (0.09)	0.36	1	Chr. 7 / 90 cM (0.09)	0.04	0	–

*r*_g_, cross-validated standardized accuracies of prediction. Cross-validation was based on data from POP-A and POP-B tested across 15 environments for grain yield (GY) and thousand kernel weight (TKW), 16 environments for test weight (TW), 11 environments for falling number (FN), and 10 environments for protein content (PC) and soluble pentosan content (SPC). Dev_ln_, natural logarithmic transformation of deviation variance; QTL, number of stable QTLs detected; Chr., chromosome; Pos., chromosomal position of the QTL detected; *R* ^2^, percentage of phenotypic variance explained by the detected QTL.

### Accuracy of prediction of phenotypic stability with genomic selection is higher than with marker-assisted selection

Applying genomic selection instead of marker-assisted selection led to considerably increased accuracies of prediction of phenotypic stability for all traits under consideration (Tablse 4 and 5). We found higher accuracies of prediction for POP-B as compared to POP-A for all phenotypic stability parameters and traits, except for the deviation variance Dev of falling number and protein content (Table 5). This discrepancy in accuracies of prediction between both populations can be explained by a narrower genetic base of the cross between the lines Lo90-N × Lo115-N (POP-A) with an average degree of polymorphisms of 45% than of the cross Lo115-N × Lo117-N (POP-B) with an average degree of polymorphisms of 67%. In addition, we obtained consistently higher accuracies of prediction for static than for dynamic stability parameters for all traits except for protein content.

**Table 5.** Cross-validated standardized accuracies of prediction for genomic selection of three phenotypic stability parameters of six traits

Trait	POP-A			POP-B		
	*r*_g_β	*r*_g_Var	*r*_g_Dev_ln_	*r*_g_β	*r*_g_Var	*r*_g_Dev_ln_
GY	0.50	0.62	0.01	0.91	1.13	0.07
TKW	0.59	0.69	0.31	0.81	0.88	0.32
TW	0.68	0.17	0.08	0.88	0.43	0.17
FN	0.75	0.82	0.04	0.52	0.62	–0.27
PC	–0.19	0.02	0.26	0.09	0.19	–0.02
SPC	0.54	0.63	0.00	0.66	0.76	0.08

*r*_g_, cross-validated standardized accuracies of prediction. Cross-validation was based on data from POP-A and POP-B tested across 15 environments for grain yield (GY) and thousand kernel weight (TKW), 16 environments for test weight (TW), 11 environments for falling number (FN), and 10 environments for protein content (PC) and soluble pentosan content (SPC). Dev_ln_, natural logarithmic transformation of deviation variance.

## Discussion

Only a very limited number of studies have so far examined the genetic architecture of phenotypic stability in plants (Kraakman *et al.*, 2004), although it is considered a central component for resilient, environmentally sound, and resource-efficient crop production systems (Finlay and Wilkinson, 1963; Francis and Kannenberg, 1978; Becker and Léon, 1988; Malosetti *et al.*, 2004). Rye is a very robust crop (Geiger and Miedaner, 1999) and, hence, an interesting model species for studying the genetics of phenotypic stability. We used genomic and extensive phenotypic data collected in up to 15 000 yield plots coming from 16 diverse environments (Fig. 1 and Supplementary Figure S1) with the main goal being to gain initial insights into the genotype–phenotype map of phenotypic stability of several important agronomic and quality traits in rye.

Following previous suggestions (Daetwyler *et al.*, 2008; Lian *et al.*, 2014), we focused on the ability to predict the performance of individuals within single bi-parental populations. This approach allows a reduction in confounding effects associated with the genetic variance among bi-parental populations (Würschum *et al.*, 2012). It is important to note that both marker-assisted and genomic selection exploit knowledge of the genetic architecture as well as being influenced by the relatedness between the individuals of the estimation and test sets (Habier *et al.*, 2007; Gowda *et al.*, 2014; Jiang *et al.*, 2014; Wang *et al.*, 2014). This has to be kept in mind while interpreting the results of the prediction accuracies, because individuals within bi-parental populations are full sibs.

### The genetic architecture of yield stability is complex with an absence of large-effect QTLs

The substantially lower estimates of heritabilities for grain yield stability as compared to the heritabilities for yield itself (Table 2, Fig. 2) clearly underline that it is more resource demanding to precisely measure yield stability in rye. Our results are in accordance with recent experimental findings on yield stability in barley (Mühleisen *et al.*, 2014). As a potential way to reduce the complexity and thus the required scale of field trials, grain yield can be deconstructed in its components such as tiller number per area, kernels per spike, and thousand kernel weight, which can also be determined precisely using high-throughput phenotyping platforms (Busemeyer *et al.*, 2013*a*, *b*). The slightly higher heritability for thousand kernel weight stability as compared to grain yield stability (Fig. 2) indicates that focusing on yield stability components is a potential alternative for indirect selection, which warrants further research. The magnitude of heritability estimates, however, was still only moderate pointing to the challenge of efficient phenotypic selection, which makes these traits an interesting target for marker-assisted or genomic selection (Lande and Thompson, 1990).

Static yield stability is negatively associated with grain yield in many crops and, consequently, varieties with pronounced yield stability often show only low yield performance (Francis and Kannenberg, 1978; Pham and Kang, 1988; Duarte and Zimmerman, 1995; Sneller *et al.*, 1997; Tollenaar and Lee, 2002; Mekbib, 2003; Mühleisen *et al.*, 2014). The static yield stability concept is therefore considered as being not economically relevant and the dynamic yield stability concept is promoted (Becker and Léon, 1988; Mühleisen *et al.*, 2014). Interestingly, grain yield was not at all associated with static yield stability in our study (Table 3). The observed discrepancy of our results with findings in other crops may be explained by an outstanding sturdiness of rye (Martis *et al.*, 2013) which is further boosted by the use of three-way hybrids exhibiting high intrinsic levels of yield stability (Becker *et al.*, 1982; Mühleisen *et al.*, 2014). Consequently, our findings indicate that static yield stability, along with dynamic yield stability, is an economically relevant parameter in rye breeding.

The absence of stable QTLs for yield stability and its component thousand kernel weight stability in our study (Table 4), and the low proportion of phenotypic variation explained by the QTLs in the cross-validation study, which also tends to be overestimated (Schüürmann *et al.*, 2008), suggests a complex genetic architecture. This observation is in line with previous results observed for the genetic architecture underlying grain yield in rye, pointing to the relevance of the infinitesimal model (Miedaner *et al.*, 2012). Genomic selection is expected to be a powerful tool for predicting the performance for such complex traits in rye (Wang *et al.*, 2014). In accordance with this anticipation, we observed moderate to high cross-validated standardized accuracies of prediction of static yield stability (Table 5). This shows that if highly precise static yield stability estimates are available, robust genomic prediction models can be developed within bi-parental populations. It is important to note, however, that even with a doubling of the phenotyping resources, predicted heritabilities for yield stability parameters would not exceed 0.6 (Supplementary Figure S2). Thus, establishment of a diverse training population for genomic selection, which is well characterized for yield stability, is very challenging and requires huge investments in multi-environmental field trials.

### Genetic architecture of phenotypic stability for quality traits in rye

For rye, the ideotype with respect to quality traits depends mainly on the intended end use (Miedaner *et al.*, 2012). For baking quality, high pentosan and low protein content should be combined with falling numbers passing 120 s and ideally ranging between 120 to 180 s (Weipert *et al.*, 1995; Geiger and Miedaner, 1999; Münzing, 2007; Kučerová, 2009). Test weight should be maximized as an indirect criterion for high starch content (Souza *et al.*, 2002; Geiger and Miedaner, 2009). In contrast, for feeding purposes pentosan content should be minimized and protein content maximized (Miedaner *et al.*, 2012). Consequently, static as well as dynamic phenotypic stabilities of quality traits are of high economic interest. We could not find any QTLs for phenotypic stability of protein content in both rye populations. No QTL was found for phenotypic stability of soluble pentosan content in population A (Table 4), which is in the agreement with QTL results for soluble pentosan content obtained by Miedaner *et al.* (2012). The lack of QTLs cannot be explained by limitations by using an indirect method to determine quality traits, because the developed calibration models were highly accurate (Table 1). Furthermore, we observed low and sometimes even negative accuracies of prediction when applying genomic selection strategies (Table 5). Thus, breeding for phenotypic stability of protein content remains challenging even if the use of advanced genomic tools is considered.

Dynamic phenotypic stability for test weight, falling number and soluble pentosan content is characterized by low heritability estimates, with an average value of 0.1 (Fig. 2). Taking this heritability and additionally the population size of 220 individuals into account, the power to detect a QTL explaining 10% of the genotypic variation even under a relaxed significance threshold will not exceed 0.2 (Charcosset and Gallais, 1996). Thus, the experimental setup empowers QTL detection underlying dynamic phenotypic stability only for QTLs exhibiting very large effects. In contrast, heritability estimates for the static phenotypic stability of test weight, falling number, and soluble pentosan content averaged up to 0.42 for the regression coefficient and 0.34 for environmental variance (Fig. 2). Considering these heritabilities and the population size, the expected power to detect a QTL explaining 10% of the genotypic variation is above 0.6 for β and above 0.5 for Var (Charcosset and Gallais, 1996). In accordance with the expected high power for detecting major QTLs with our experimental settings, we observed robust QTLs for static phenotypic stability of test weight, falling number, and soluble pentosan content, which were detected in more than 50% of the cross-validation runs (Table 4). The reliability of the QTL for static phenotypic stability of falling number was also reinforced by the fact that it has been detected in both populations. Thus, this QTL is an interesting candidate for further fine mapping.

The static stability for falling number in POP-B was significantly correlated with falling number values in an undesired direction (Table 3). In line with these findings, a QTL for falling number had been previously detected on chromosome 6 (Miedaner *et al.*, 2012) close to the region where we observed the QTL underlying static stability for falling number. A more detailed inspection of the genomic region revealed, however, that the marker proximal to the QTL for static stability of falling number was not significantly associated with falling number as such (*P* values of 0.88 and 0.64 for β in POP-A and POP-B, respectively; *P* values of 0.45 and 0.64 for Var in POP-A and POP-B, separately). Consequently, marker-assisted selection for static stability of falling number based on the QTL located on chromosome 6 does not impair the selection of varieties with high falling number.

Beyond the prospect of marker-assisted selection within these two connected bi-parental populations, genomic selection holds even greater potential for improving the static phenotypic stability of test weight, falling number, and soluble pentosan content (Table 5). Integrating information on stable QTLs in the genomic selection approaches opens up options for further improving the accuracy of prediction. This has been shown recently for genomics-assisted improvement of plant height and heading time in wheat (Zhao *et al.*, 2014) and also represents an interesting method for improving the phenotypic stability of quality traits in rye.

### Conclusion

Phenotypic stability of varieties is pivotal for combating climate change-related challenges. Our findings reveal that large-scale multi-environmental phenotyping is needed to efficiently select for enhanced phenotypic stability. Genome-assisted breeding approaches offer the potential for building prediction models upon such extensive phenotypic data, facilitating an economic improvement of phenotypic stability in crops.

## Supplementary material

Supplementary data can be found at *JXB* online.

Supplementary Table S1. Conditional probabilities of QTL genotypes of population Lo115-N × Lo117-N and population Lo115-N × Lo90-N.

Supplementary Table S2. Marker data of 220 F_3:4_ test-cross progenies of segregating rye population Lo115-N × Lo90-N used for genomic selection.

Supplementary Table S3. Marker data of 220 F_3:4_ test-cross progenies of segregating rye population Lo115-N × Lo117-N used for genomic selection.

Supplementary Table S4. BLUEs for test-cross progenies across environments and estimates of phenotypic stability parameters for six traits in population Lo115-N × Lo90-N and Lo115-N × Lo117-N.

Supplementary Figure S1. Dendrogram of cluster analysis including 15 environments for thousand kernel weight (TKW), 16 environments for test weight (TW), 11 environments for falling number (FN), and 10 environments for protein content (PC) and soluble pentosan content (SPC) for POP-A and POP-B.

Supplementary Figure S2. Predicted heritability of the three stability parameters Dev, β, and Var for the quantitative traits grain yield (GY) and thousand kernel weight (TKW), and for the quality traits test weight (TW), falling number (FN), protein content (PC), and soluble pentosan content (SPC) based on an increasing number of test environments in POP-A and POP-B.

## Funding

This research was conducted within the project ‘Erweiterung der genetischen Basis von Hybridroggen für Korn- und Biomasseleistung sowie Trockenheitstoleranz mittels Mehrlinienkartierung und DH-Technik’ financially supported by the German Federal Ministry of Food and Agriculture via the ‘Fachagentur Nachwachsende Rohstoffe e.V.’, Gülzow, Germany (Grant ID: 22021711).

## Supplementary Material

Supplementary Data
