# Supplementary material for: First insights into the genotype–phenotype map of phenotypic stability in rye
Source: J Exp Bot. 2015 Apr 6;66(11):3275–84. doi: 10.1093/jxb/erv145 (PMC4449549; doi:10.1093/jxb/erv145)
Supplement: Supplementary Data [file supp_66_11_3275__index.html]

First insights into the genotype–phenotype map of phenotypic stability in rye — First insights into the genotype–phenotype map of phenotypic stability in rye — Supplementary Data 

# First insights into the genotype–phenotype map of phenotypic stability in rye

## Supplementary Data

Data files

**Files in this Data Supplement:**

- Supplementary Data - Supplementary Data
- Supplementary Data - Supplementary Data
- Supplementary Data - Supplementary Data
- Supplementary Data - Supplementary Data
- Supplementary Data - Supplementary Data
