# Supplementary figures and images for: First insights into the genotype–phenotype map of phenotypic stability in rye
Source: J Exp Bot. 2015 Apr 6;66(11):3275–84. doi: 10.1093/jxb/erv145 (PMC4449549; doi:10.1093/jxb/erv145)

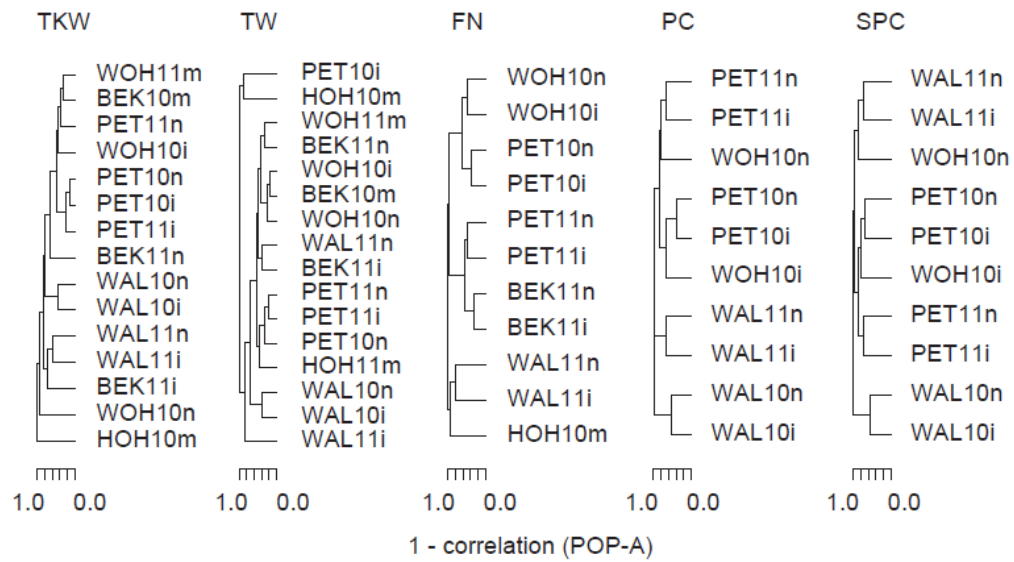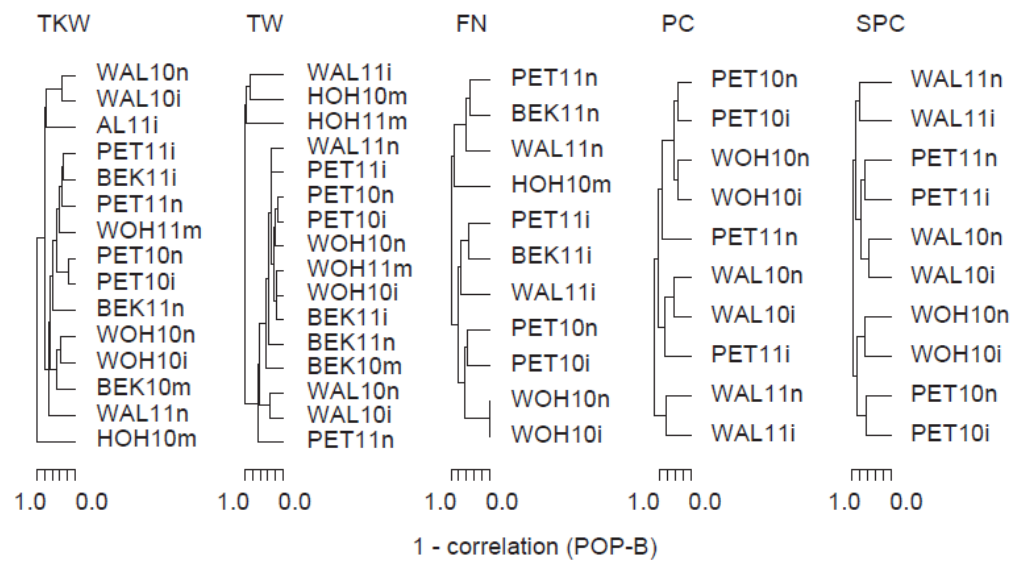

Wang et al. Figure S1

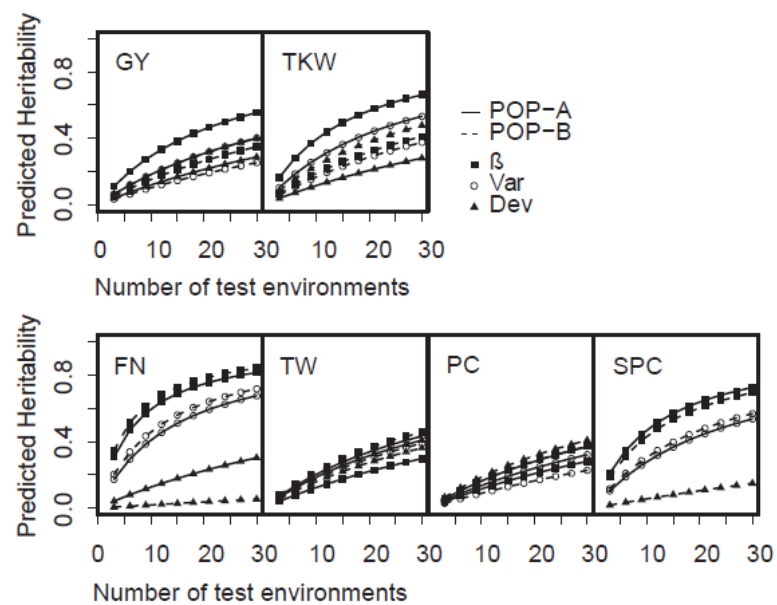

Wang et al. Figure S2

Supplement: Supplementary Data [file supp_erv145_jexbot135137_file005.pdf]
